# Poisonous substances used to capture and kill the greater cane rat (*Thryonomys swinderianus*)

**DOI:** 10.1002/vms3.259

**Published:** 2020-03-31

**Authors:** Edward K. Essuman, Kingsley K. Duah

**Affiliations:** ^1^ Department of Nutrition and Dietetics University of Health and Allied Sciences Ho Ghana; ^2^ Faculty of Agricultural Education University of Education Winneba Ghana; ^3^ Osei Tutu Senior High School Kumasi Ghana

**Keywords:** akrantie, carbofuran, grasscutter, poisonous substance, yellow oleander

## Abstract

The greater cane rat, as it is commonly known, is often called grasscutter (in Ghana, Nigeria and other regions of West Africa). Even though is highly patronized as a delicacy by a majority of Ghanaians (akrantie—Twi language) mostly in the rural areas, the persistent reports on people being poisoned as a result of eating food prepared with grasscutter which has been captured/killed by the use of poison are deterring people from consuming the grasscutter meat despite its high protein content. The objective of this study was, therefore, to investigate the actual ingredients that are used in the formulation of poison to capture grasscutter for human consumption. Questionnaires were administered to participants (farmers) who are involved in grasscutter hunting to solicit the ingredient they formulate to poison the grasscutter in their hunting. To prove the activeness of these ingredients, the main ingredient used in formulating the poison to capture the grasscutters were tested on two male grasscutters and these were yellow oleander root (*Cascabela thevetia*; syn: *Thevetia peruviana*) powder and carbofuran. The findings of the experimental trial revealed that the grasscutter that was fed with yellow oleander root powder did not die but showed some signs of intoxication and staggered each time it tried to move. However, the grasscutter fed with carbofuran died within 10 hr of being poisoned. Majority of the participants attested to the fact that the use of poison increases their chance of capturing the grasscutter, especially in the dry season since the poison is not washed away by the rainwater. However, consuming grasscutter poisoned with either yellow oleander root power or carbofuran could be detrimental to human health.

## Graphical abstract

Consuming grasscutter poisoned with either yellow oleander root power or carbofuran could be detrimental to our health. There is a need to increase public awareness.

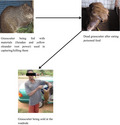

## 1 INTRODUCTION

It has been reported that in Africa, bushmeat may form over 80% of the consumption of animal protein of some countries (Ntiamoah‐Baidu, 1998). Asibey (1974) and Ntiamoah‐Baidu (1998) indicated that the carcasses of the grasscutters contributed about 75% by weight of game meat sales in Kantamanto market in Accra, Ghana, for a period of 25 years. In some local market about 73 tons of grasscutter meat can be sold within a year (Opara, 2010). The high patronage of grasscutter meat resulted in the inclusion of grasscutter on the export trade of Ghana by the Ghana Export Promotion Council (GEPC) as reported by Asibey (1974).

Unfortunately, the contribution of bushmeat protein to human diets is endangering species survival. The rapid destruction of the natural habitats through agriculture, mining, logging, bushfires and the indiscriminate hunting of these animals through crude and unscrupulous methods such as deliberate poisoning and bush burning due to the high demand for bushmeat are threats to the extinction of these animals (Adamu, 1993). The crude and unscrupulous methods such as poison used in capturing bushmeat not only poses a threat to the extinction of the animals but also poses serious health problems like stomach disorders, diarrhoea, abdominal cramps, nausea and vomiting, abdominal distension and excessive gas production, headache and fever to the consumers.

In order, therefore, to protect the contribution made by bushmeat to the human diets and also reduce poverty and malnutrition in rural areas, prudent measures need to be adopted to curb unscrupulous methods of meat hunting and to step up the production of some non‐traditional animals such as grasscutters due to its high patronage as a delicacy and contribution to the national economy. This research work aims at identifying the substances used in the formulation of poison to capture and kill grasscutter for human consumption.

## 2 MATERIALS AND METHODS

### 2.1 Study site

The research study was conducted in the Amansie West District of the Ashanti region of Ghana, where the youth are noted for grasscutter hunting.

### 2.2 Sources and type of data

Primary data were mainly used for the study, and these were obtained from grasscutter hunters. In all, 10 villages in the district (Essuowin, Awherewa, Mosesso, Asaase, Atwere, Antoakurom, Manso‐Nkwanta, Daatano, Tontonkurom and Ankam) were randomly selected using convenient sampling technique with 20 participants from each village. Primary qualitative data were collected via observation.

### 2.3 Data collection

The data were collected through personal interviews with the use of a semi‐structured questionnaire, alongside discussions and direct observations. The interviews were conducted with the help of trained field assistants who speaks the native language of the people. The questionnaire was used to obtain data on the ingredients used in formulating poisonous substances to capture grasscutters.

### 2.4 Experimental trials

An experiment on the chemicals (poisonous substance) used in capturing the grasscutters was tested on two male grasscutters. The materials used in formulating the poison were obtained from Essuowin in the Amansie West District of the Ashanti Region, while the formulation and the administration of the poison took place at the Non‐Traditional Section of the University of Education, Winneba, Mampong Ashanti Campus—Ghana.

**Figure 1:**
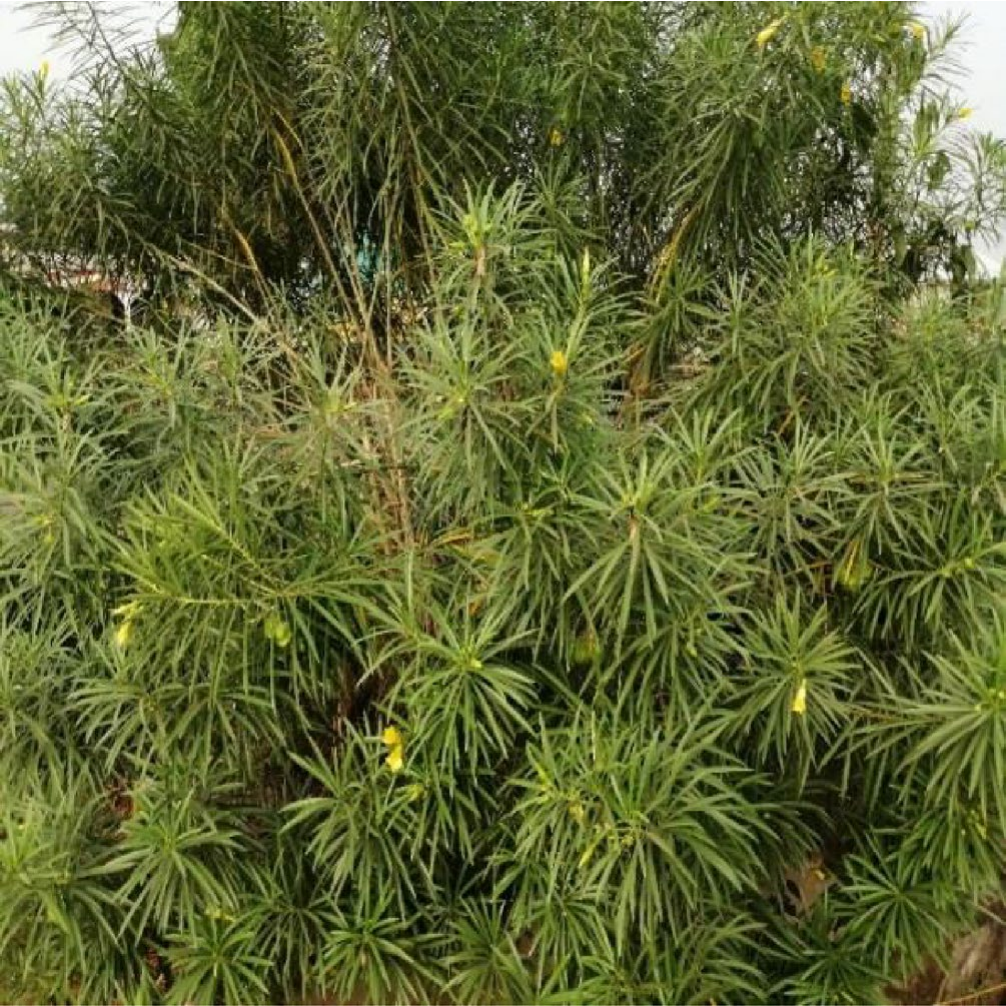
Yellow oleander (*Thevetia peruviana*) plant

**Figure 2:**
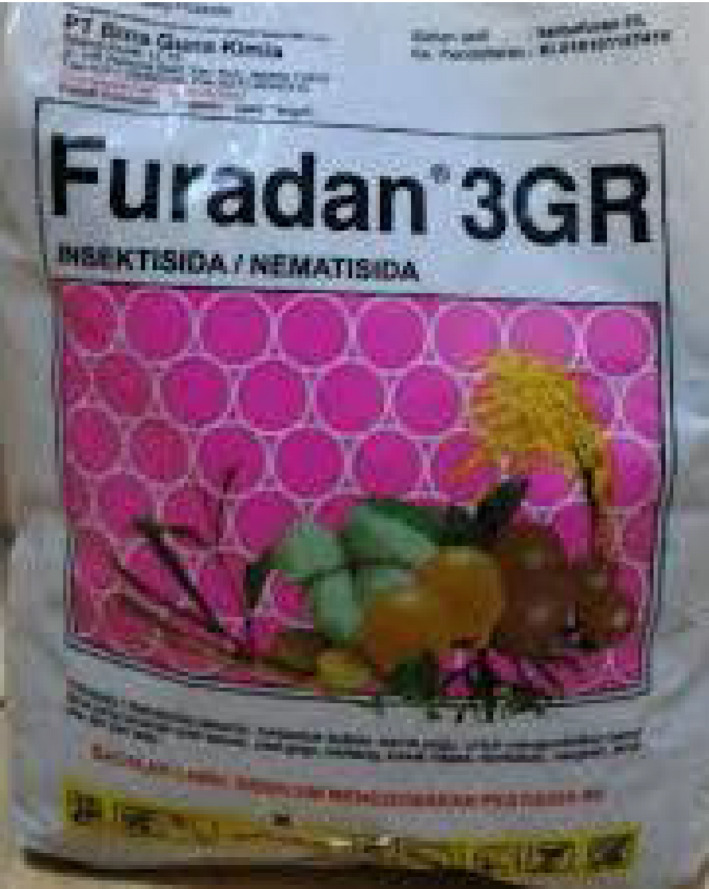
Furadan (carbofuran)

Since the main ingredient used in the formulation of the poison to feed the grasscutter was either the root of the plant of yellow oleander (Figure 1) or carbofuran (Figure 2), an incision was made through the stem of the forage (*Pennisetum purpureum*) mostly used to feed the grasscutter and about 8 g of carbofuran was placed in it and fed to the grasscutter (9‐months old, weighing 3.9 kg) in its cage. Another forage (*Pennisetum purpureum*) was also dipped in water and about 10 g of the powered yellow oleander root was smeared on it and fed to another male grasscutter in its cage. The grasscutters consumed the forage including the poison on them leaving a very small portion in the case.

### 2.5 Statistical analysis

All analyses were performed using the Statistical Package for the Social Sciences (SPSS for Windows, release 14.0, 2005). Descriptive analysis of data was done using frequency tables and pie chart.

## 3 RESULTS

### 3.1 Materials used by farmers in formulating the poison to capture grasscutters

The results of the interview revealed that the materials shown in Table 1 are often used by the majority of the farmers in formulating the poisonous substance to capture grasscutters. Few occasionally add sleeping pills (zaleplon or zolpidem) and broken bottles to the materials.

**Table 1:** Materials commonly used in formulating poison to catch grasscutters by farmers/hunters in Amansie West District of Ghana

Materials	Estimated quantities
Yellow oleander (roots) powder (*Thevetia peruviana*)	200.8 g
Human male urine	1.5 L
Common salt (NaCl)	200.0 g
Furadan (carbofuran)	24.6 g
Stone (moulded soil) or corn	—
Sleeping pills	—
Broken bottles	—

### 3.2 Preparation and administration of the poisonous substance by farmers/grasscutter hunters

Table 1 shows the materials and the quantities that are used by the hunters in formulating the poison in the fields. About 1.5 L of the urine is stored for three (3) days to give a pungent smell followed by the addition of 200 g common salt to form a solution. Carbofuran is then added to the solution and stirred to obtain a uniform mixture. An alternative poison formulation containing yellow oleander roots with the bark removed is also used. The cambium region is scraped from the main root, dried for about 7 days and ground to powder. The yellow oleander root powder is then mixed with human male urine (that has been stored for 3 days). Salt is then added to make a solution (poison). The quantities given by the farmers were estimated as some said they use more than that in their preparation of the poison substance.

The poison substance is then used as bait and set as a trap at a grazing site for grasscutters.

The soil at the site is hallowed with a cutlass or a hoe and the solution is uniformly spread over the hallowed soil. Some of the participants also stated that, instead of using the solution, they sometimes mix the carbofuran with soil and spread it at the sites where grasscutters are found grazing. Stone is then heated to become red‐hot and put on the hollowed soil and the urine‐salt solution is poured over the red‐hot stone and made to cover the entire area where the carbofuran has been spread. The red‐hot stone helps to diffuse the scent of the urine to the surroundings to attract the grasscutters. In some situations, farmers mix either the carbofuran or the yellow oleander root powder with soil to form a ball of soil and then dry it in the sun to cake. The ball of poisoned‐soil mixture which is caked then serves as the bait or trap. A number of them are made and deposited at the site where the grasscutters are fond of grazing.

### 3.3 Usage of poisonous substances by the participants in capturing/killing grasscutter

The villages and the number of people who often use poisonous substances in capturing grasscutters are shown in Table 2. Ahwerewa recorded the highest number of people found of using poisonous substances to capture grasscutters, followed by Ankam. The study also confirms that grasscutter is a delicacy in this group of questioned Ghanaians. It was revealed that 50% of the participants (Table 3) preferred grasscutter above other meats. All those interviewed preferred game meats over other types.

**Table 2:** Usage of poisonous substances in capturing grasscutters in villages in Amansie West District of Ghana

Village	Number of participants	Participants who use poison
Awherewa	20	8
Ankam	20	7
Essuowin	20	6
Moseaso	20	6
Asaase	20	6
Daatano	20	5
Atwere	20	5
Tontonkurom	20	4
Antoakurom	20	4
Manso‐Nkwanta	20	4
Total	200	55

**Table 3:** Choice of game meat patronized by participants

Game meat	Number of participants	Percentage (%)
Grasscutter	100	50.0
Rat	25	12.5
Antelope	25	12.5
Antelope/Duiker	20	10.0
Bush baby	10	5.0
Hedgehog	5	2.5
Partridge	5	2.5
Ground squirrel	5	2.5
Deer	5	2.5
Total	200	100

### 3.4 Effects of the poison on the grasscutters after consuming the forage containing the poison often used by the farmers

After setting the bait using the elephant grass, the grasscutter that ate the bait containing the yellow oleander root powder did not die but showed signs of intoxication that included ataxia and somnolence. The poison lasted approximately 18 hr (consumed toxin at 1:53 p.m. and was still intoxicated until 08:20 AM the following day) and complete recovery took 72 hr from ingestion.

In contrast, the grasscutter fed with carbofuran died approximately 10 hr after ingestion. Before death it displayed signs that included arching of the mouth and neck onto the cage floor, sneezing, laboured breathing, increased salivation, anorexia, prostration, muscle tremors and protrusion of the third eyelid. Signs were evident approximately 10 min after ingestion.

### 3.5 Season/time for setting poisoned baits to capture grasscutters

**Figure 3:**
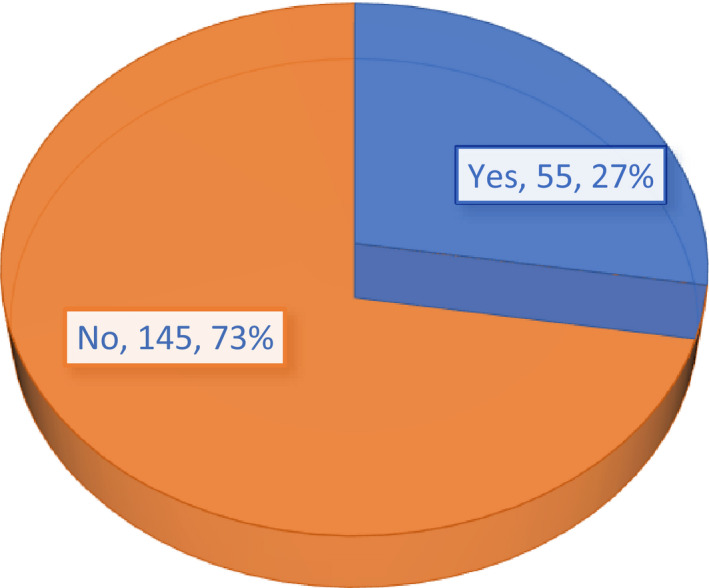
The use of poison as bait to trap grasscutters by farmers

Out of the two hundred (200) participants, 55 reported using poison to bait wild grasscutters (Figure 3). Of these (55), fifty‐one (51) of them representing about 92.7% said they set the poisonous substances during the dry season since in the raining season, water may enter the bait, dilute the poison thereby rendering it less active. However, the remaining four (4) people representing about 7.3% said they apply the bait (poisonous substance) during the raining season and they attributed their reason to the fact that it is during that time that the grasscutters come to destroy their farm produce.

### 3.6 Usage of grasscutters by farmers after capturing/killing them

Fifty people (50) representing about 90.9% of the participants who agreed to the use of poison to capture/kill the grasscutter said they sell the grasscutters captured with the poison, especially when they are many. The remaining 5, representing 9.1%, said they eat the grasscutters captured with the poisonous substances even when they capture many of them.

### 3.7 Sale of grasscutter captured/killed with poisonous substances

Out of the 50 respondents, who said they sell the grasscutters captured with the poisonous substance, 40 of them, representing 80%, said they sell them to the ‘chop bar’ operators (food vendors). They added that they make them aware of the mechanism they use in capturing them so that they will remove the viscera making sure that, the contents do not make contact with the carcass before using it to prepare food for sale.

Five (5) of the remaining 10, representing 10%, said they sell them to recognized game meat lovers in the town while the remaining five (5) people also said they sell them by the roadside to people in passing vehicles.

## 4 DISCUSSION

### 4.1 Materials use in capturing or killing grasscutters

Yellow oleander and carbofuran are the main ingredients used in preparing poisonous substances to bait grasscutters. Yellow oleander is a plant with all its parts containing toxic cardiac glycosides, like neriifolin, thevetin A, thevetin B and oleandrin, which are lethal after ingestion. Plants with this self‐poisoning effect are common in subtropical and tropical parts of the world (Prasad et al., 2016). Furadan, on the other hand, is the trade name of the most toxic carbamate pesticides called carbofuran which is used to control insects in a wide variety of field crops.

However, in this study, the majority of the participants reported using carbofuran in formulating poisonous substance to poison grasscutters mostly during the dry season to avoid rain washing off the poison. This finding is supported by Ahmad, Walgenbach, and Sutter (1979) who stated that persistence of carbofuran in soils is a function of many factors, including pesticide formulation, rate and method of application, soil type, pH, rainfall, temperature moisture content and microbial populations. This implies that carbofuran, which is the main chemical in the bait is strongly affected by water hence not advisable to be used as a bait in the rainy season. Most of the respondents also said that more animals are captured when baited during the dry season since during those dried periods food is scarce and this makes the animals eat anything they come into contact with.

Few of the participants use the poisonous substances during the raining seasons to deter the grasscutters from destroying their farm produce like maize, sugar‐cane, cassava, rice, yam, etc. as said by Addo‐Quaye, Saah, and Tachie‐Menson (1993) about the feeding habit of grasscutter in causing considerable damage to cultivated crops. This suggests that these groups of people do not practice the use of poisonous substances in hunting grasscutter as a business; rather they use it as a means of protecting their farm produce. Therefore, the actual time in which these farmers set their bait is during the dry season.

Besides the materials mostly used by the farmers/hunters to capture or kill grasscutters, some also add sleeping pills (zaleplon or zolpidem) and broken bottles to the mixture. The addition of the sleep pills is to hasten the grasscutter to doze off. Zolpidem is one of the most commonly prescribed sleep pills worldwide (Kripke, Langer, & Kline, 2012). Another research also confirmed zolpidem usage for short term management of poor sleep quality among professional firefighters (Mehrdad, Haghighi, & Esfahani, 2015).

### 4.2 Health effect of consuming grasscutter poisoned with these substances

The experimental trial of ingestion of these materials for capturing or killing grasscutters caused the grasscutters to display signs that are reported in larger mammals that were exposed to carbofuran (Palmer & Schlinke, 1973). A similar observation was reported by the New Jersey Department of Health and Senior Services (2005) that exposure to carbofuran can cause weakness, sweating, nausea and vomiting, abdominal pain and blurred vision in humans. Larger concentrations can cause muscle twitching, loss of coordination and may cause breathing to stop when inhaled or with skin contact.

Grasscutter which is part of game meat was preferred by all the participants and this finding is in line with research conducted by Tutu, Ntiamoa‐Baidu, and Asuming‐Brempong (1993) which stated that within the West Africa sub‐region grasscutter is the favourite bush meat species sold in markets. Most grasscutter hunters easily get access to the carbofuran due to its widespread use in agriculture as insecticides, hence contamination of food, water and air has become imminent and consequently, adverse health effects are inevitable in humans, animals, wildlife and fish (Gupta, 1995).

The captured grasscutters with the poisonous substances are sold or eaten by the farmers. Thus, obtaining their animal protein from the grasscutter meat as stated by Ntiamoah‐Badu (1998) that a sizable amount of the animal protein in the diet comes from the wild as bush meat which is patronized by a majority of Ghanaians mostly in the rural areas. However, grasscutter captured or killed using poisonous substances makes the content of the viscera as poisonous as the poison itself. This is in line with research conducted by Yiadom (2007) who reported that the internal organs/ viscera must not be eaten but must be buried in pits because if not properly disposed of, any animal that eats it will die. However, the stomach content of the grasscutter (the content of the small intestine) is used to flavour soup. Many people are of the view that eating grasscutter without the addition of the stomach content in the soup causes it to lose its taste and delicacy.

The major problem here has been one's ability to determine the genuineness of the mechanism from which the grasscutters were captured as it is very difficult to distinguish between grasscutter killed with a gun from those poisoned and shot with a gun. This is because some hunters intentionally shoot the grasscutters after capturing them with the poisonous substance, whereas others accidentally shoot the grasscutters which are somnolent due to the poison, but have staggered away from where the poisonous substance was placed and take them as unpoisoned meat killed by gunshot only. These data show that everyone who consumes grasscutter is at risk from secondary poisoning, whatever the source of meat.

## 5 CONCLUSION

The result of the experiment indicates that poisonous substances used to capture and kill cane rats or grasscutters can have a serious health effect on the lives of the consumers. This is because yellow oleander and carbofuran are known to be lethal after ingestion. However, from the information gathered, the best way of avoiding one's self from being poisoned by eating the grasscutter meat is to remove the entire digestive system from the oesophagus to the rectum and making sure that the content of the intestine does not spill on the carcass. This practice becomes necessary especially when one purchases grasscutter meat as it is impossible to know whether it has been poisoned or shot.

## CONFLICT OF INTEREST

There is no conflict of interest among the authors.

## AUTHOR CONTRIBUTION

**Edward Ken Essuman:** Conceptualization; Data curation; Formal analysis; Investigation; Methodology; Resources; Software; Writing‐original draft; Writing‐review & editing. **Kingsley Karikari Duah:** Conceptualization; Data curation; Formal analysis; Investigation; Methodology; Resources; Supervision; Validation; Visualization; Writing‐original draft; Writing‐review & editing.

## ETHICAL STATEMENT

The research was approved by the Institutional Ethical Committee at the University of Education, Winneba.
